# Identification of microRNA-16-5p and microRNA-21-5p in feces as potential noninvasive biomarkers for inflammatory bowel disease

**DOI:** 10.18632/aging.202428

**Published:** 2021-02-01

**Authors:** Rui Zhou, Peishan Qiu, Haizhou Wang, Huijie Yang, Xueying Yang, Mingliang Ye, Fan Wang, Qiu Zhao

**Affiliations:** 1 Department of Gastroenterology, Zhongnan Hospital of Wuhan University, Wuhan 430071, China; 2 Hubei Clinical Center and Key Laboratory of Intestinal and Colorectal Diseases, Wuhan 430071, China; 3 Department of Medical Records, The Central Hospital of Enshi Autonomous Prefecture, Enshi 445000, China

**Keywords:** microRNA, feces, IBD, ulcerative colitis, Crohn’s disease

## Abstract

Background: Inflammatory bowel disease (IBD) is a chronic idiopathic gastrointestinal disease. Increasing evidence suggests that microRNAs (miRNAs) may participate in the pathophysiology of IBD.

Methods: A miRCURY™ LNA Array and *in situ* hybridization were employed to screen for differentially expressed miRNAs (DEMs) in fecal specimens from 41 IBD patients (22 ulcerative colitis (UC), 19 Crohn’s disease (CD)) and 23 healthy controls (HC). RT-qPCR was performed to confirm the findings. The DEMs target genes and corresponding biological functions were predicted by bioinformatics analysis.

Results: Compared with HC, miR-16-5p in the feces was up-regulated both in UC and CD patients (p < 0.01), while miR-21-5p was up-regulated only in UC patients (p < 0.01). TargetScan 7.2, miRWalk, and miRDB were used to predict 216 public target genes of miR-16-5p and miR-21-5p, and six hub genes (PIK3R1, GRB2, SUZ12, NTRK2, Smurf2, and WWP1) were analyzed using the STRING database and Cytoscape. All the hub genes promote the occurrence and development of IBD-related colorectal cancer.

Conclusions: The elevated levels of miR-16-5p and miR-21-5p in feces of IBD patients have to guide significance for the noninvasive clinical diagnosis of IBD and have a warning effect on the occurrence of IBD-related colorectal cancer.

## INTRODUCTION

Inflammatory bowel disease (IBD) is a cluster of chronic idiopathic, immune, relieving, and recurrent gastrointestinal diseases that occur when genetically susceptible populations are exposed to environmental risk factors. IBD includes Crohn’s disease (CD) and ulcerative colitis (UC) based on differences in clinical phenotypes [1]. The etiology of IBD is considered as complex interactions among genes, immune responses, and environmental factors [2]. The medical and surgical treatment of IBD is primarily driven by an accurate assessment of disease activity. The mucosal inspection via endoscopy remains the golden standard for estimating disease activity in IBD, and the use of cross-sectional imaging is increasing [3]. However, most of these monitoring methods are not suitable and optimal for routine clinical applications due to their disadvantages of intrusiveness, time-consuming, and expensive. It is urgently needed to develop better tools to overcome this dilemma in screening and disease activity assessment of IBD.

In recent years, studies on noninvasive measurable biomarkers for the diagnosis of IBD have gradually increased [4, 5]. Due to the influence of bewildering factors such as age, sex, or body mass index, conventional inflammatory marks (such as C-reactive protein (CRP) and erythrocyte sedimentation rate) do not accurately reflect disease activity [6]. Most importantly, both of them are not specific to IBD [7]. Due to the complexity of detection technology, the detection results lack clinical reliability, lack of assessment of responsiveness to changes in disease states, and cannot replace the performance of endoscopy assessment. Fecal levels of calprotectin and lactoferrin have not been identified as biomarkers for diagnosing IBD disease activity [8]. Fecal molecular aberration detection is also a promising noninvasive method for IBD screening, among which DNA testing is the most established test [9–11]. Johnson et al. reported significantly higher methylation rates of bone morphogenic protein 3 (BMP3) or N-Myc downstream-regulated gene 4 (NDRG4) in IBD lesions than matched controls, which revealed that specific DNA markers that were present in advanced IBD neoplasia could be detected in the tissues and feces when small adenomas occur in IBD patients [9].

Epigenetic factors can mediate interactions between the environment and the genome [12]. As a primary epigenetic mechanism, RNA interference delivered by microRNA (miRNA) may have a significant effect on the pathogenesis of IBD and other diseases [13–16]. Koukos et al. found that miR-4284 was the most significantly down-regulated microRNA in the intestinal mucosa of pediatric patients with UC compared with non-IBD controls. What's more, the lower the expression level of miR-4284 predicted the higher the disease activity of IBD [13]. MiRNAs, a group of short single-stranded RNA molecules, could reduce gene expression through the deterioration of target mRNAs or blockage of translation [17], thereby altering the export of many protein-coding genes and related pivotal cellular biological functions [18]. MiRNAs were reported to be associated with the regulation of autophagy, inflammation, and fibrosis associated with IBD [14–16]. Besides, the normal intestinal development of mice is inseparable from the role of miRNAs produced by intestinal epithelial cells [19]. The most widely used method for screening miRNAs in whole blood, serum specimens, and intestinal mucosa as biomarkers of IBD disease activity are microarray approaches, which are rarely used in feces [20, 21]. Fecal miRNAs are acquired in a relatively minimally invasive manner and can be briskly quantified by quantitative polymerase chain reaction (qPCR) or microarrays. It follows that fecal miRNAs are alluring noninvasive biomarkers for the diagnosis of IBD. In this study, the fecal miRNA expression profile from IBD patients and healthy controls (HC) were compared to recognize novel potential miRNA biomarkers with higher sensitivity involved in IBD in feces. Our study will help to avoid invasive radiological or endoscopic investigation in IBD patients and provide a direction for the exploration of new treatment strategies.

## RESULTS

### MiRNA expression profiling in IBD feces

To explore the underlying role of fecal miRNAs in diagnosing IBD, fecal miRNA expression profiles of IBD patients and healthy volunteers were evaluated by microarray analysis. Microarray analysis revealed 3100 miRNAs were detected in total in the fecal specimens collected from 41 IBD patients (22 UC and 19 CD) and 23 HC. Of seven DEMs identified in the feces of IBD patients, two miRNAs (let-7i-3p and miR-326) exhibited decreased expression, and five miRNAs (miR-15a-5p, miR-16-5p, miR-21-5p, miR-338-5p, miR-483-5p) demonstrated increased expression compared with that in the feces of HC (fold change > 7, P < 0.05), as shown in Table 1.

**Table 1:** Differentially expressed miRNAs in the feces of IBD patients detected by miRCURY™ LNA Array (fold change > 7, P < 0.05).

**Upregulated miRNA**	**Downregulated miRNA**
miR-15a-5p	let-7i-3p
miR-16-5p	miR-326
miR-21-5p	
miR-338-5p	
miR-483-5p	

### Validation of miRNA candidates in IBD feces

**Figure 1:**
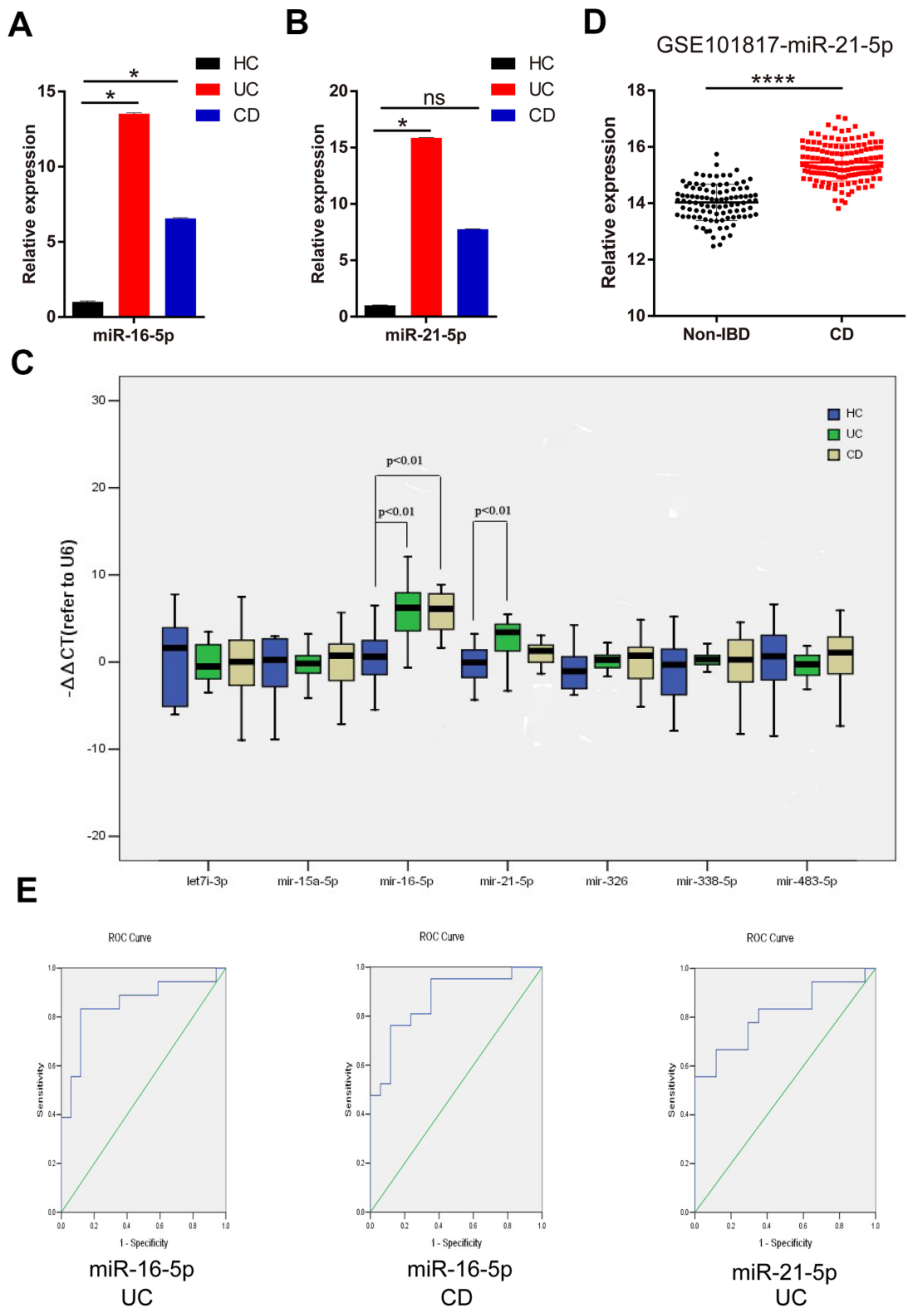
**MiR-21-5p and miR-16-5p are potential noninvasive feces markers for IBD.** (**A**) Expression of miR-16-5p in fecal specimens from HC, UC, and CD. (**B**) Expression of miR-21-5p in fecal specimens from HC, UC, and CD. (**C**) Expression of seven candidate DEMs (let-7i-3p, miR-15a-5p, miR-16-5p, miR-21-5p, miR-326, miR-338-5p and miR-483-5p) in fecal specimens from HC, UC and CD. (**D**) Compared with normal intestinal mucosa tissue based on GSE101817, the expression of miR-21-5p in the intestinal mucosa of CD patients was up-regulated. (**E**) Diagnostic value of miR-16-5p and miR-21-5p in fecal specimens of IBD patients was evaluated by ROC curves. *p < 0.05, **p < 0.01, ***p < 0.001, ****p < 0.0001; HC, healthy control; UC, ulcerative colitis; CD, Crohn’s disease; ns, not statistically significant; ROC, receiver operating characteristic.

The expression levels of DEMs in the feces of IBD patients and healthy volunteers were verified by RT-qPCR. Compared with HC, the expression of miR-16-5p was significantly increased both in the fecal specimens of UC and CD patients (Figure 1A). However, the increase of miR-21-5p was only observed in the feces of UC patients (Figure 1B), and there was no difference between CD patients and HC. It was worth noting that the expression levels of the other five miRNAs had negligible differences in fecal specimens from IBD patients and healthy volunteers (Figure 1C). Based on all these findings, the follow-up analysis of this study focused on miR-16-5p and miR-21-5p.

### Validation of DEMs

In the GSE101817 test set, miR-21-5p expression was increased in the intestinal mucosa of CD patients than in the normal intestinal mucosa (Figure 1D). Thus, in IBD patients, miR-21-5p was distinctly expressed not only in the feces but also in the intestinal mucosa.

### MiR-21-5p and miR-16-5p are potential noninvasive feces markers for IBD

The diagnostic capability of miR-21-5p and miR-16-5p for IBD was verified by Receiver operating characteristics (ROC) curves analysis. It was surprising to find that miR-16-5p showed a great power in diagnosing CD (area under curve (AUC) = 0.868, p < 0.001) and UC (AUC = 0.853, p < 0.001). Besides, the ROC curve analysis of miR-21-5p for the diagnostic accuracy of UC showed an AUC of 0.810 (p < 0.001) (Figure 1E).

### Prediction of the target genes of miR-21-5p and miR-16-5p

**Figure 2:**
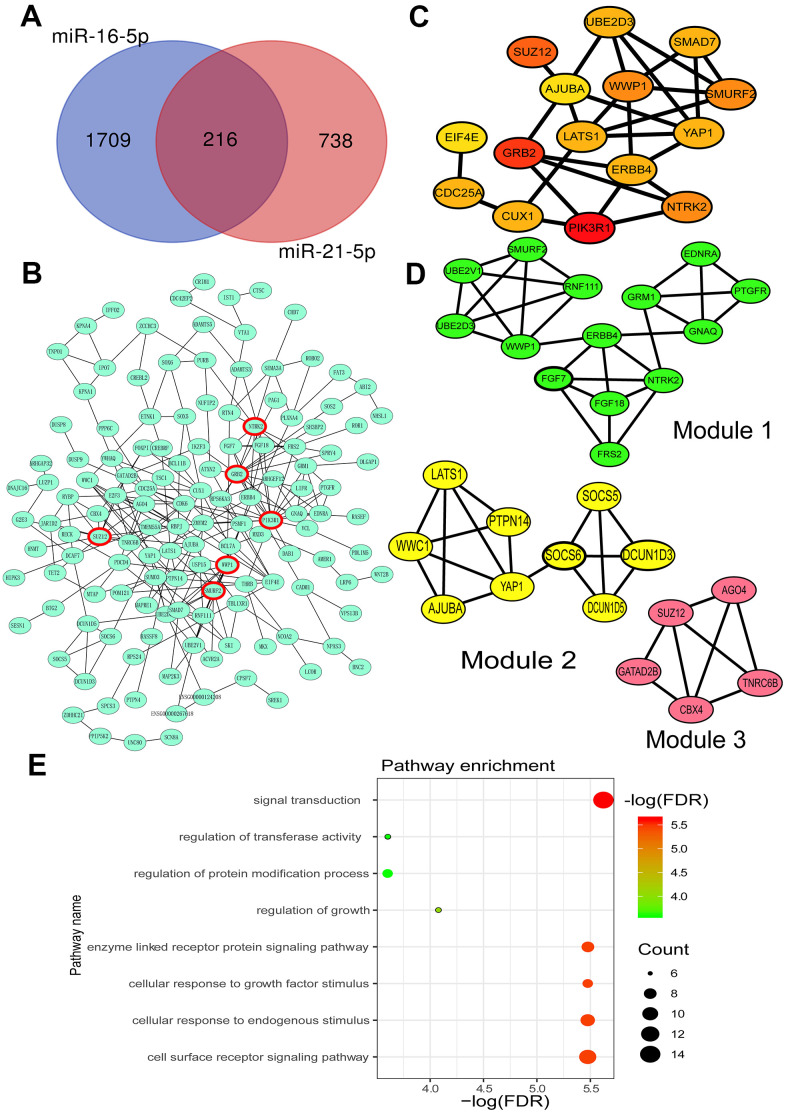
**Prediction of miR-16-5p and miR-21-5p target genes and their biological functions.** (**A**) Venn Diagram of the common target genes of miR-16-5p and miR-21-5p predicted by TargetScan, miRWalk and miRDB. (**B**) Protein-protein interaction (PPI) network of miR-21-5p and miR-16-5p target genes. (**C**) Top 15 hub target genes identified by the Cytohubba plug-in of Cytoscape. (**D**) Three key modules of the PPI network identified by the MCODE plug-in of Cytoscape. (**E**) Function analysis of all target genes in three modules. FDR, false discovery rate.

The potential target genes of miR-16-5p and miR-21-5p were obtained from three online databases (TargetScan 7.2 [22], miRWalk [23], and miRDB [24]), respectively. After duplicates removal, there were 1925 and 954 target genes of miR-16-5p and miR-21-5p, respectively. A total of 216 genes were recognized as common targets of both miR-16-5p and miR-21-5p by overlapping analysis, and the result was shown using a Venn diagram (Figure 2A).

### PPI network construction and module analysis

The Search Tool for the Retrieval of Interacting Genes (STRING) database [25] was performed to construct the protein-protein interaction (PPI) network and analyze the interaction between target genes (Figure 2B). Cytohubba, a plug-in of the STRING database, was used to find the top 15 hub genes [26] (Figure 2C). Furthermore, with degree ≥ 10 as the threshold, six genes were selected for further analysis, including phosphoinositide-3-kinase regulatory subunit 1 (PIK3R1), growth factor receptor-bound protein 2 (GRB2), SUZ12 polycomb repressive complex 2 subunit (SUZ12), neurotrophic receptor tyrosine kinase 2 (NTRK2), SMAD specific E3 ubiquitin-protein ligase 2 (Smurf2), and WW domain-containing E3 ubiquitin-protein ligase 1 (WWP1). MCODE was used to select three remarkable modules from the PPI network complex on the basis of the degree of importance [27] (Figure 2D).

### Function and pathway enrichment analysis

The function analysis of target genes in the modules was predicted using the STRING database. Kyoto Encyclopedia of Genes and Genomes (KEGG) [28] and Gene Ontology (GO) enrichment analysis of hub genes in the most notable modules showed that the crucial enrichment pathways included signal transduction, cellular response to endogenous stimulus, cellular response to growth factor stimulus, enzyme-linked receptor protein signaling pathway, cell surface receptor signaling pathway, regulation of growth, regulation of transferase activity, and regulation of protein modification process (Figure 2E). Most of these pathways involved epithelial regeneration and epithelial-mesenchymal transition (EMT) associated with IBD [29, 30].

## DISCUSSION

The etiology of IBD, a chronic inflammatory gastrointestinal disease, is unknown [31]. Over the past few decades, the incidence of IBD has increased significantly in many countries, which has placed broad social and economic pressures on public health structures and systems [32, 33]. Many studies have shown that miRNAs, as gene expression regulators, are associated with various inflammatory states [34–36]. Of course, IBD is no exception. In recent years, the key role of miRNAs in regulating the pathological progression of IBD has been gradually reported [37–40]. It has been reported that the serum miRNAs expression profiles in CD and UC patients were distinct from that of normal controls [41, 42]. Chen et al. found a positive correlation between serum miR-146b-5p expression and IBD disease activity. Furthermore, miR-146b-5p was more specific than CRP [43], which was the presently available succedaneous biomarker for inflammation in IBD [44]. Besides, Ge et al. reported that the expression of miR-125a in the inflamed intestinal mucosa of IBD patients was lower than that in healthy volunteers, and miR-125a could protect intestinal mucosa from inflammatory injury [45]. Compared with mucosal miRNA, detecting the differential expression of fecal miRNAs can more accurately assess the disease activity or mucosal healing of gastrointestinal diseases. Since differentially expressed miRNAs in feces are another manifestation of local variation, such as exfoliation of intestinal epithelial cells, exosome production, and mucosal barrier changes [40, 46]. Moreover, fecal miRNAs are relatively stable and resistant to harsh conditions, making it clinically possible as a noninvasive biomarker [46]. However, there have been relatively few studies on miRNAs screening in feces of IBD patients [47]. Duran-Sanchon et al. believed that the elevated levels of miR421, miR27a-3p, and hemoglobin in feces could identify patients with advanced adenoma or colorectal cancer (CRC) more accurately than the concentration of hemoglobin in feces alone [48].

In our study, we observed differences in fecal miRNA between IBD patients and healthy subjects through microarray approaches. The results showed that 34 miRNAs increased and nine miRNAs decreased in both UC and CD patients. Subsequently, combined with previous reports [47], we identified seven miRNAs for later studies. However, qPCR analysis of fecal specimens confirmed that miR-16-5p was increased in both UC and CD, while miR-21-5p was only increased in UC patients’ feces. Also, miR-16-5p expression was down-regulated in CRC tissues compared with the normal intestinal mucosa [49], and miR-16-5p showed sharp tumor-suppressive roles in CRC [50]. It is worth mentioning that colitis-associated CRC in IBD patients is substantially clinically different from the fragmented CRC observed in the common population. Occult evolution occurs long before clinically detectable neoplasms develop to IBD-associated carcinogenesis. [51].

MiR-21-5p was greatly obviously expressed in colorectal cancer tissues and could target tumor suppressor genes through an epigenetic modification to promote survival and resist apoptosis. Therefore, miR-21-5p was considered to be a carcinogenic miRNA [30, 52]. Moreover, miR-21-5p had higher accuracy in distinguishing superficial and deep tumors in oral squamous cell carcinoma [53]. In patients with colorectal cancer, serum miR-21-5p was higher before the surgery but down-regulated after the surgery, which revealed that the decline of miR-21-5p might support a better overall survival in colorectal cancer patients [54]. In our study, miR-21-5p was expressed remarkably only in fecal specimens of patients with UC. The important thing was that there was an inextricable relationship between UC and CRC [55]. Therefore, the increase of miR-21-5p promoted the revolution from UC to CRC.

Three online websites (TargetScan 7.2, miRWalk, and miRDB) were used to predict the potential target genes of miR-16-5p and miR-21-5p, and co-expression network construction and module analysis were carried out in Cytoscape, and six hub genes were finally screened. Among them, PIK3R1 was reported to be involved in immune-related mechanisms in the progression of UC [56]. Early embryos of mice with complete deletion of GRB2 cannot survive. Members of the GRB2 protein family are essential for the initiation and development of a variety of developmental and disease-related signaling complexes. Besides, GRB2 makes a great difference in the progression of autoimmune diseases due to its involvement in T cells’ development [57]. GRB2 associated binding protein 2/3 (Gab2/3) double knockout mice could develop spontaneous colitis with rectal prolapse and diarrhea, mainly involving macrophages and CD8^+^ T cells, which was due to the role of Gab2/3 in suppressing the inactivation of immune cells in the process of inflammation [58]. SUZ12 was reported to be an oncogene in colorectal cancer, which plays a cancer-promoting role by methylating histone H3 [59]. Compared with sporadic CRC, the expression of NTRK2 in UC-associated CRCs was significantly different [60]. The expression of Smurf2 in CRC tissues was markedly increased than the corresponding healthy intestinal mucosa, and the level of Smurf2 in microsatellite instability (MSI) CRC was also dramatically higher than that in microsatellite stability (MSS) CRC [61]. More importantly, the high expression of Smurf2 in tumors indicated a poor prognosis. Besides, the overall survival and disease-free survival of CRC patients with high WWP1 expressions were worse than those with low WWP1 expressions [62], which was consistent with the tumorigenic effect of Smurf2. In short, most hub target genes of miR-16-5p and miR-21-5p are essential for the pathomechanism and development of CRC. Patients with UC or CD have a significantly increased risk of gastrointestinal and extra-intestinal malignancies, with CRC being the most common cancer associated with IBD [63]. Mortality from cardiovascular disease, infection, and cancer in IBD patients increases year by year [64]. Active cancers are the second leading cause of mortality after cardiovascular disease in IBD patients [65].

In conclusion, fecal specimens were collected from clinical IBD patients, and differentially expressed miRNAs associated with the occurrence of IBD were screened. The elevated levels of miR-16-5p and miR-21-5p in feces of IBD patients are of guiding significance for the noninvasive clinical diagnosis of IBD and have a warning effect on the occurrence of IBD-related CRC in IBD patients.

## MATERIALS AND METHODS

### Patient recruitment

Fecal specimens were collected from 41 IBD patients and 23 healthy subjects. Table 2 listed the basic characteristics of all subjects. Before specimen collection, written informed consent was acquired from all subjects, and the study protocol was accepted by the Ethics Review Committee of Zhongnan Hospital of Wuhan University (Hubei, China). All authors and researchers involved in the study read and strictly adhered to the ethics guidelines of the World Medical Association (Declaration of Helsinki). The healthy controls had no abnormal symptoms and a negative colonoscopy, excluding those with other gastrointestinal diseases or taking antibiotics. The subjects were between the ages of 16 and 58.

**Table 2:** Clinical characteristics of the study subjects.

**Category**	**UC**	**CD**	**HC**	**P value**
No. of cases	22	19	23	-
Age at enrollment, y				
Mean±SD	36.22±10.11	34.19±11.76	29.71±10.08	ns
Gender, number (%)				
Female	5(22.7)	12(63.2)	7(30.4)	
Disease duration, mo	41.11±43.51	55.64±71.63		ns
Disease location, n (%)				
L1	-	9(47)		
L2	-	7(37)		
L3	-	3(16)		
Disease extent, n (%)				
Proctosigmoiditis	5(23)	-		
Left-sided colitis	6(27)	-		
Pancolitis	11(50)	-		
CRP (mg/L)	6.05±7.46	17.57±18.13	-	ns
ESR (mm/h)	14.67±11.12	30.33±28.52	-	ns
ALB (g/L)	41.94±3.78	38.64±4.45	-	ns
WBC (×10^9^/L)	6.82±2.72	6.09±2.71	-	ns
HGB (g/L)	128.33±17.96	102.52±14.23	-	ns
RBC (×10^12^/L)	4.11±0.58	3.73±0.76	-	ns
HCT (%)	35.41±9.96	30.69±4.17	-	ns
PLT(×10^9^/L)	222.71±37.90	288.14±109.08	-	****
FOBT (+), n (%)	11(50.0)	7(36.8)	-	-

UC, ulcerative colitis; CD, Crohn’s disease; HC, healthy control; SD, standard deviation; CRP, C-reactive protein; ESR, erythrocyte sedimentation rate; ALB, albumin; WBC, white blood cell; HGB, hemoglobin; RBC, red blood cell; HCT, hematocrit; PLT, platelet count; FOBT, fecal occult blood test; *p < 0.05, **p < 0.01, ***p < 0.001, ****p < 0.0001; ns, not statistically significant.

### Fecal specimen collection and fecal miRNA extraction

Fresh human fecal specimens (20-30 g) were collected with a 50-ml specimen cup and stored at -80° C for a long time. All samples were tested together after collection to reduce the batch effect. 10 mg feces (wet weight) from each specimen was added into a 1.5 ml RNase-free microcentrifuge tube containing 1 ml of Trizol LS reagent (Invitrogen, Carlsbad, CA, USA). Use an RNase-free grinder to homogenize the feces mixture completely. 200 μl of chloroform was added to the microcentrifuge tube for RNA precipitation. According to the manufacturer’s recommendation, QIAgen miRNeasy Mini Kit (Qiagen, Valencia, CA, USA) was used for total RNA extraction. Total RNA was eluted in RNase-free water. Nanodrop 2000 (Thermo Fisher Scientific, Waltham, MA, USA) was used for the assessment of total RNA concentration and standardized the total RNA concentration of all fecal samples to 1ng/ul.

### MiRNA microarray screening

Kang Chen (Shanghai, China) performed the miRNA microarray screening after RNA isolation from the specimens. Total miRNAs from fecal specimens were labeled with miRCURY™ Hy3™/Hy5™ fluorescent dye using the miRCURY™ Power Labelling Kit (Exiqon, Vedbaek, Denmark) following the manufacturer’s guidelines, and then hybridized to the miRCURY™ LNA miRNA Array (version 18.0, Exiqon, Vedbaek, Denmark). The Axon GenePix 4000B microarray scanner (Molecular Devices, USA) was used to scan the slides. The Axon GenePix 4000B microarray scanner (Molecular Devices, USA) was used to scan the slides. The data were extracted with Agilent Feature Extraction v10.7 software and analyzed with GeneSpring GX (version 13.0, Agilent Technologies, California, USA). After normalization, the absolute value of the fold change of gene expression was more than seven as a significant difference, and this method was used to screen DEMs.

### Quantitative reverse transcriptase-polymerase chain reaction (qRT-PCR) detection

The miRNA expression level was assessed utilizing qRT-PCR, which was performed using the SYBR green method on Bio-Rad according to the manufacturer’s specification. The RNAs were subjected to reverse transcribed with TaqMan miRNA Reverse Transcription kit (Applied Biosystems, Foster City, CA, USA) following the protocol, obtaining cDNA product. All amplifications were performed in triplicate, and the quantification of the results was analyzed by the comparative Ct (2^-ΔΔ^Ct) method. U6 was used to standardize miRNA expressions.

### Validation of DEMs

The GSE101817 downloaded from the GEO database (http://www.ncbi.nlm.nih.gov/geo/) was used as a test set to confirm the relationship between the previously selected miRNAs and IBD.

### Predicting the target gene of DEMs

The possible target genes of miRNAs were predicted using TargetScan 7.2 [22] (http://www.targetscan.org/vert_72/), miRWalk [23] (http://mirwalk.umm.uni-heidelberg.de/), and miRDB [24] (http://www.mirdb.org/). These databases contain experimentally validated miRNA targets and provide the most recently updated targets. The anticipated target genes of each differentially expressed miRNA from three different sites were combined. After removing the duplicates, the Venn diagram was used for overlap analysis.

### Protein-protein interaction (PPI) network and module analysis

The PPI network analysis of the overlapping target genes was performed by the STRING (http://string-db.org, version 11.0) [25] online database. The minimum required interaction score of > 0.4 was defined as statistically significant. Cytoscape (version 3.7.2) enabled the visualization of complex protein interaction networks [66]. The Molecular Complex Detection (MCODE) (version 1.5.1) was an application for performing module analysis in Cytoscape [27]. The PPI network was built in Cytoscape and used MCODE to identify the most significant modules. Set the thresholds as follows: degree cut-off = 2, node score cut-off = 0.2, Max. depth = 100 and k-score = 2. Further hub gene screening was performed using the cytoHubba (version 0.1) plug-in of Cytoscape [26].

### Function and pathway enrichment analysis

Gene Ontology (GO) contains three aspects of functional information, including the biological processes involved in genes, the location of cells, and the molecular functions they play and organizes these functional concepts into a DAG (Directed Acyclic Graph) structure [67]. KEGG (https://www.kegg.jp/) is an exhaustive database of functional interpretation and practical application of genomic information and integrates macromolecular datasets from genome sequencing and other high-throughput experimental techniques [28]. KEGG and GO functional enrichment analysis for target genes was performed using the STRING database [25]. False discovery rate (FDR) < 0.05 was defined to be statistically significant.

### Statistical analysis

Statistical analyses were achieved by GraphPad Prism 5.0 (GraphPad Software, Inc.). The mean and standard deviation (SD) or standard deviation (SE) are used to describe the values obtained by each continuous variable in the study. For categorical variables, absolute and relative frequencies were used. The nonparametric correlation between the two variables was evaluated by the Spearman rank correlation coefficient (r). The diagnostic effect of fecal miRNAs compared to the control group for IBD were analyzed by ROC curve and estimated by AUC. P < 0.05 was considered statistically significant.
